# An on-line processing strategy for head movement interferences removal of dynamic brain electrical impedance tomography based on wavelet decomposition

**DOI:** 10.1186/s12938-019-0668-8

**Published:** 2019-05-09

**Authors:** Ge Zhang, Weichen Li, Hang Ma, Xuechao Liu, Meng Dai, Canhua Xu, Haoting Li, Xiuzhen Dong, Xingwang Sun, Feng Fu

**Affiliations:** 1 Department of Radiology, Bethune International Peace Hospital, Shijiazhuang, China; 2 Department of Biomedical Engineering, Fourth Military Medical University, Xi’an, China

**Keywords:** Brain electrical impedance tomography, Head movement, Wavelet decomposition, Gaussian distribution, Brick-laying algorithm

## Abstract

**Background:**

Head movement interferences are a common problem during prolonged dynamic brain electrical impedance tomography (EIT) clinical monitoring. Head movement interferences mainly originate from body movements of patients and nursing procedures performed by medical staff, etc. These body movements will lead to variation in boundary voltage signals, which affects image reconstruction.

**Methods:**

This study employed a data preprocessing method based on wavelet decomposition to inhibit head movement interferences in brain EIT data. Mixed Gaussian models were applied to describe the distribution characteristics of brain EIT data. We identified head movement signal through the differences in distribution characteristics of corresponding wavelet decomposition coefficients between head movement artifacts and normal signals, and then managed the contaminated data with improved on-line wavelet processing methods.

**Results:**

To validate the efficacy of the method, simulated signal experiments and human data experiments were performed. In the simulation experiment, the simulated movement artifact was significantly reduced and data quality was improved with indicators’ increase in PRD and correlation coefficient. Human data experiments demonstrated that this method effectively suppressed head movement in signals and reduce artifacts resulting from head movement artifacts in images.

**Conclusion:**

In this paper, we proposed an on-line strategy to manage the head movement interferences from the brain EIT data based on the distribution characteristics of wavelet coefficients. Our strategy is capable of reducing the movement interference in the data and improving the reconstructed images. This work would improve the clinical practicability of brain EIT and contribute to its further promotion.

## Background

Dynamic brain electrical impedance tomography (EIT) is a non-invasive, low-cost, continuous monitoring functional imaging technology used in the biomedical field, which has applications for early diagnosis of cerebrovascular disease. Usually, the EIT system uses 16 electrodes placed uniformly on the head to apply safe currents and measures boundary voltage at two different instants, then reconstructs intracranial impedance changes between the two instants according to Acerta in algorithm [1, 2]. To ensure that their constructed image is accurate, the boundary voltage variation between the reference frame and current frame should originate from changes in intracranial impedance. Thus, good and stable connection between electrodes and the scalp is a crucial factor for image monitoring of brain EIT. However, dynamic brain EIT monitoring is a long-term process. The change of connection status is a common occurrence during our previous clinical studies because of factors like: patient’s body movements (head rotation, body turning) and nursing procedures performed by medical staff. These body movements lead to head movements and change the electrode–skin contact status, which introduce movement interferences in data collection and image reconstruction [3, 4]. Therefore, there is an urgent need for appropriate methods to process the movement interferences if we want to further promote the dynamic brain EIT research.

Currently, the researches of brain EIT mainly focus on employing EIT for neurological functional studies through simulation experiments and animal models [5, 6]. This makes few reports on head movement processing for long-term brain EIT monitoring. There are some studies related to the analysis and processing of clinical body movement interferences in lung EIT. Lozano et al. [7] analyzed errors in data collected from prolonged dynamic EIT clinical monitoring and quantitatively evaluated the effects of changes in electrode position and patient posture on measurement data. Subsequently, Adler and Guardo [8] analyzed the effects caused by changes in electrode position due to lung expansion and contraction during breathing. Following that, Adler [4] used maximum a posteriori (MAP), a posteriori estimation method for correction of data errors during severe body movement interferences (i.e., detached electrodes). Based on the MAP method, Asfaw and Hartinger employed simulation and reciprocity methods, respectively, for testing of problematic electrodes to obtain a priori information of compensatory electrode data [3, 9]. Recently, Zhang used a weighted correlation coefficient method for testing multiple problematic electrodes caused by body movements, and employed data from grey model predictions for compensatory processing [10]. However, the methods above are all used in situations with extreme body movement interference such as electrode disconnection. The affected measurements are impossible to restore by data processing, which is a different problem compared with that we need to solve in this work. Besides, as the monitoring cycle for lung EIT is relatively short, the problem of body movement interference during breathing in EIT is not as urgent as during brain EIT [11, 12]. Goren et al. [13] reported EIT data with noises and movement artifacts in stroke patients. But their data were collected through multifrequency EIT method, which was different from our time-difference EIT method. Besides, the EIT data contained movement artifacts which were just rejected instead of being processed, which could not offer us help in the movement interferences management.

Impedance mapping, EEG, near-infrared spectroscopy, and other similar techniques also encounter body movement interference problems during clinical practice. Several methods for movement interference removal in such electrical signals have been reported. These methods can be mainly classified into two types.

The first type is based on adaptive filters. In this occasion, additional input signals are required for movement artifacts removal. Researchers acquired the expected signal or interference by set extra measurement channel in the hardware [14–16].

The second type mainly includes Wiener filter methods, signal-related improvement methods and Kalman filter method, which does not need additional input. The wiener filter requires the acquisition of a priori information of expected signals, like signal’s power spectrum [17]. The signal improvement method was proposed by Cui et al. [18]. The disadvantage is that the expected signal and interference should be maximally negatively correlated if possible. The Kalman filter method requires a priori information models in which noise distribution is obtained [19]. Besides, there are also the principle component analysis (PCA) method [20], independent component analysis (ICA) method [21], spline interpolation method [22], etc., which all have particular scope of application and limitations in real-time processing. Therefore, these methods are not ready for removal of movement interferences in dynamic brain EIT.

Wavelet decomposition is a signal processing method that analyzes the time–frequency characteristics of signals to detect and process body movement interference components. As there are differences in amplitude and duration between head movement interferences and normal photoelectric signals, these differences could be distinguished and managed in wavelet domain. Wavelet method shows good performance when processing weak signals, and provides us with referential application in dynamic brain EIT. However, the literature reporting use of this method does not mention specific real-time processing [14, 23–25].

Based on the points above, our study took advantage of the differences in the distribution characteristics of brain EIT signals and movement interference signals in wavelet domain to extract and process the movement interference signals by employing event probabilities. Meanwhile, brick-laying algorithm strategies were used to achieve on-line operation of this processing strategy.

In this scenario, the patients suffer from brain injury and have to stay lying on the sickbed in supine position. They will keep the supine position after any initiative or passive head movement for comfort or medical consideration. The status of affected electrodes will be switched between being pressed and released. This kind of movement interferences may not result in data acquisition failure, but contaminate the brain EIT signal with spike-like artifacts. The spike-type movement interferences are the objects to manage in our study.

## Methods

### EIT data acquisition procedures

**Fig. 1:**
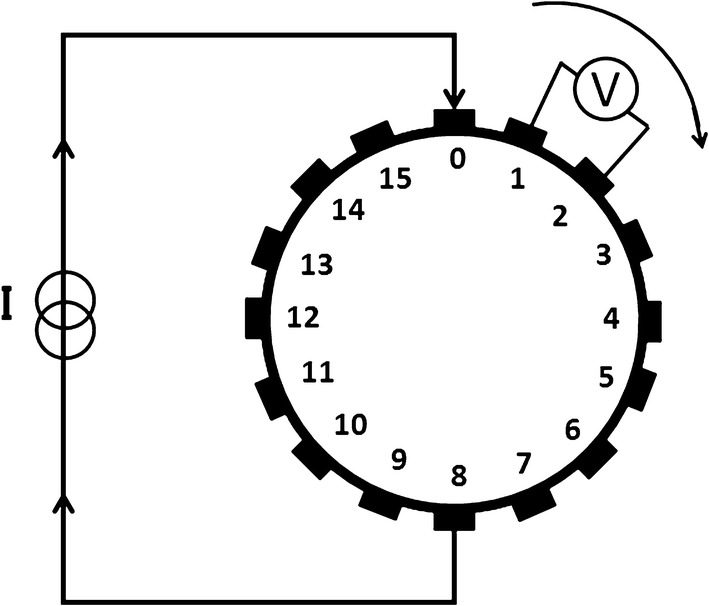
Illustration of opposite-excitation adjacent-measurement data acquisition protocol of the brain EIT system. The current flows into the circular field through electrode 0 and flows out through opposite electrode 8. Voltages are measured on the other adjacent electrodes

The brain EIT system used requires the annular attachment of 16 equidistant electrodes on the subject’s head. Opposite excitation and adjacent acquisition protocol were adopted to measure boundary voltage data. The data acquisition process is shown in Fig. 1. In one completed at a acquisition process, electrodes 0 and 8 were used as current entry and exit electrodes, respectively, while electrodes 0–1, 1–2, 2–3…15–0 were used as measurement electrode pairs to obtain 16 sets of voltage differences. The above steps were repeated by injecting by injecting the current through electrode pair 1–9, 2–10,…, 15–7 sequentially and all adjacent measurements constituted one frame of data (256 sets) [26]. While the measurements adjacent to the excitation electrode contain more contact impedance than internal impedance and need to be discarded during reconstruction, there are $16 \times ( 1 6 - 4) = 192$ valid measurements in each frame. Therefore, there are 192 data channels that require observation and processing [27].

### Influences of head movement interferences on boundary voltage

The EIT image reconstruction process can be briefly summarized as:

1 $$ y = Bx $$

where $x$ represents input data, which is a frame of boundary voltage data, $y$ represents the conductivity distribution of the target field, and $B$ is the construction matrix which is determined by the finite element model (FEM) of the target field, and is closely related to the number of model elements, number of boundary measurement values, background conductivity, electrode model, etc.

With respect to dynamic EIT, the reconstruction process can be briefly described as:

2 $$ y = B(x_{\text{f}} - x_{\text{b}} ) $$

where $x_{\text{f}}$ represents the boundary voltage measurement data of the current frame and $x_{\text{b}}$ represents the boundary voltage measurement data of the reference frame. Under normal circumstances, the assumption is that differences in $x_{\text{f}}$ and $x_{\text{b}}$ only originate from changes in intracranial impedance.

When the electrode contacts the scalp, electrode–scalp contact impedance unbalance occurs [28, 29]. During brain EIT detection, contact impedance affects measurements in two ways: first, when electrodes are used as the measurement tool, the presence of electrode–scalp contact impedance is equivalent to the addition of a conductive layer outside the normal field. It will affect current distribution beneath the electrode. In addition, if contact impedance is sufficiently large, it will affect the common-mode rejection ratio at the measurement end [30, 31]. Second, the contact impedance may decrease the injected current when the electrodes act as excitation pair. Therefore, the head movement leads to artifacts into EIT signal [32]. These contact impedance variations only occur in the current frame and result in image artifacts. Since the movement duration is shorter than the voltage changes caused by intracranial pathophysiological changes, it is characterized by rapid changes in EIT signal. And due to the high resistivity of the skull compared to the degree of intracranial impedance changes, the ratio of corresponding boundary voltage changes is far smaller than the changes in intracranial impedance. Thus, it can be concluded that the head movement disrupts the continuity of EIT signals, which is equivalent to high-frequency outlier in stable signals. This makes it possible to use wavelet analysis methods to discriminate normal signals and from head movement interferences.

### Processing strategy for head movement interferences based on wavelet decomposition

In previous analyses, the movement interference signals are considered as sudden changes with larger amplitude values in stationary signal. In contrast, slowly changing boundary voltage signals caused by internal impedance changes are regarded as low-frequency signals. In the wavelet domain, the low-frequency components correspond to wavelet coefficients in smaller values and the high-frequency components correspond to wavelet coefficients in larger values. So we can discriminate normal signals from movement interferences in temporal manner through wavelet decomposition [33, 34]. Especially, in the scenarios where normal components and interferences are aliasing in frequency domain, the wavelet strategy is able to exert its time–frequency analysis features and achieve better results.

Original signal $s(t)$ can be expressed as:

3 $$ s(t) = s_{0} (t) + \varepsilon (t) $$

where $s_{0} (t)$ represents EIT signals without head movement interferences and $\varepsilon (t)$ represents head movement interference signal. The Mallet method was used to carry out wavelet decomposition, and raw signals can be expressed as [35, 36]:

4 $$ s(t) = \sum\limits_{k} {v_{{j_{0} k}} \phi_{{j_{0} k}} (t) + \sum\limits_{{j = j_{0} }} {\sum\limits_{k} {w_{jk} \psi_{jk} (t)} } } $$

where $\phi_{jk} (t) = 2^{j/2} \phi (2^{j} t - k)$ represents the function of the reconstruction scale, $\psi_{jk} (t) = 2^{j/2} \psi (2^{j} t - k)$ represents the reconstructed wavelet function, $j$ and $k$ represent the number of layers and scale translation coefficient in wavelet decomposition, respectively, $v$ represents the scale decomposition coefficient, and $w_{j}$ represents the wavelet decomposition coefficient at the $j$th level. In addition,

5 $$ w_{jk} = \sum\limits_{l} {g(l - 2k)v_{j + 1} (l)} $$

6 $$ v_{jk} = \sum\limits_{l} {h(l - 2k)v_{j + 1} (l)} $$

where $j = j_{0} , \ldots ,J - 1,$ $k = 0, \ldots ,2^{J - 1} ,$ $g(l - 2k)$ is a high-pass filter, and $h(l - 2k)$ is a low-pass filter. The corresponding wavelet coefficient at the first level is:

7 $$ w_{1k} = \sum\limits_{l} {g(l - 2k)s(l)} $$

8 $$ v_{1k} = \sum\limits_{l} {h(l - 2k)s(l)} $$

Combining the above methods, the corresponding decomposition coefficient can be written as:

9 $$ w_{1k} = w_{{1k,s_{0} }} + w_{1k,\varepsilon } $$

10 $$ v_{1k} = v_{{1k,s_{0} }} + v_{1k,\varepsilon } $$

The coefficients for various layers in top-down decomposition coefficient can be expressed as:

11 $$ w_{jk} = w_{{jk,s_{0} }} + w_{jk,\varepsilon } $$

12 $$ v_{jk} = v_{{jk,s_{0} }} + v_{jk,\varepsilon } $$

The movement interference management based on wavelet method is achieved by dealing with the detailed wavelet coefficients. The normal brain EIT signals are continuous and relatively smooth and slow-changing [37–39]. Compared with the head movement interferences, the corresponding wavelet decomposition coefficients are small fluctuations concentrated around zero. This characteristic can be used to carry out discrimination and processing head movement interference signals. We adopted Molavi’s wavelet processing strategy to manage the movement interferences [23].

Once a segment of signal is decomposed by wavelet function, the wavelet coefficients can be represented by a Gaussian mixture model containing two zero-mean Gaussian distributions [40]. One Gaussian distribution describes large wavelet decomposition coefficients, while the other Gaussian distribution describes the remaining small coefficients. Among these coefficients, larger coefficients correspond to dramatic signal changes and the corresponding Gaussian distribution is relatively dispersed. Then the large coefficients can be set to zero to inhibit dramatic changes in signals. Giving the probability density function of Gaussian distribution with smaller variation, we can calculate the geometric probability of wavelet coefficients. We define rejection probability to present this geometric probability. Considering the wavelet coefficient $w_{{jk,s_{0} }}$ of signals can be represented by $w_{{jk,s_{0} }} \sim N(0,\sigma^{2} ),$ the definition of rejection probability is:

13 $$ p_{jk} = \left( {\phi \left( {\frac{{\left| {w_{jk} } \right|}}{{\hat{\sigma }}}} \right) - 0.5} \right)*2 $$

where $\phi (k) = \frac{1}{{\hat{\sigma }\sqrt {2\pi } }}\int_{ - \infty }^{k} {e^{{\frac{{ - k^{2} }}{{2\hat{\sigma }^{2} }}}} } {\text{d}}k$ is the Gaussian probability density function. If the wavelet decomposition coefficient does not originate from the Gaussian distribution with smaller variation, the calculated rejection probability should be higher than the ones that comply with this distribution. We can set threshold $\alpha$ to pick out and manage the wavelet coefficients of head movement interferences. The management strategy is:

14 $$ w_{jk} = \left\{ {\begin{array}{*{20}l} 0 \\ {w_{jk} } \\ \end{array} } \right.\begin{array}{*{20}l} {\quad {\text{if }}\;p_{jk} > \alpha } \\ {\quad {\text{if}}\; \, p_{jk} < \alpha } \\ \end{array} $$

**Fig. 2:**
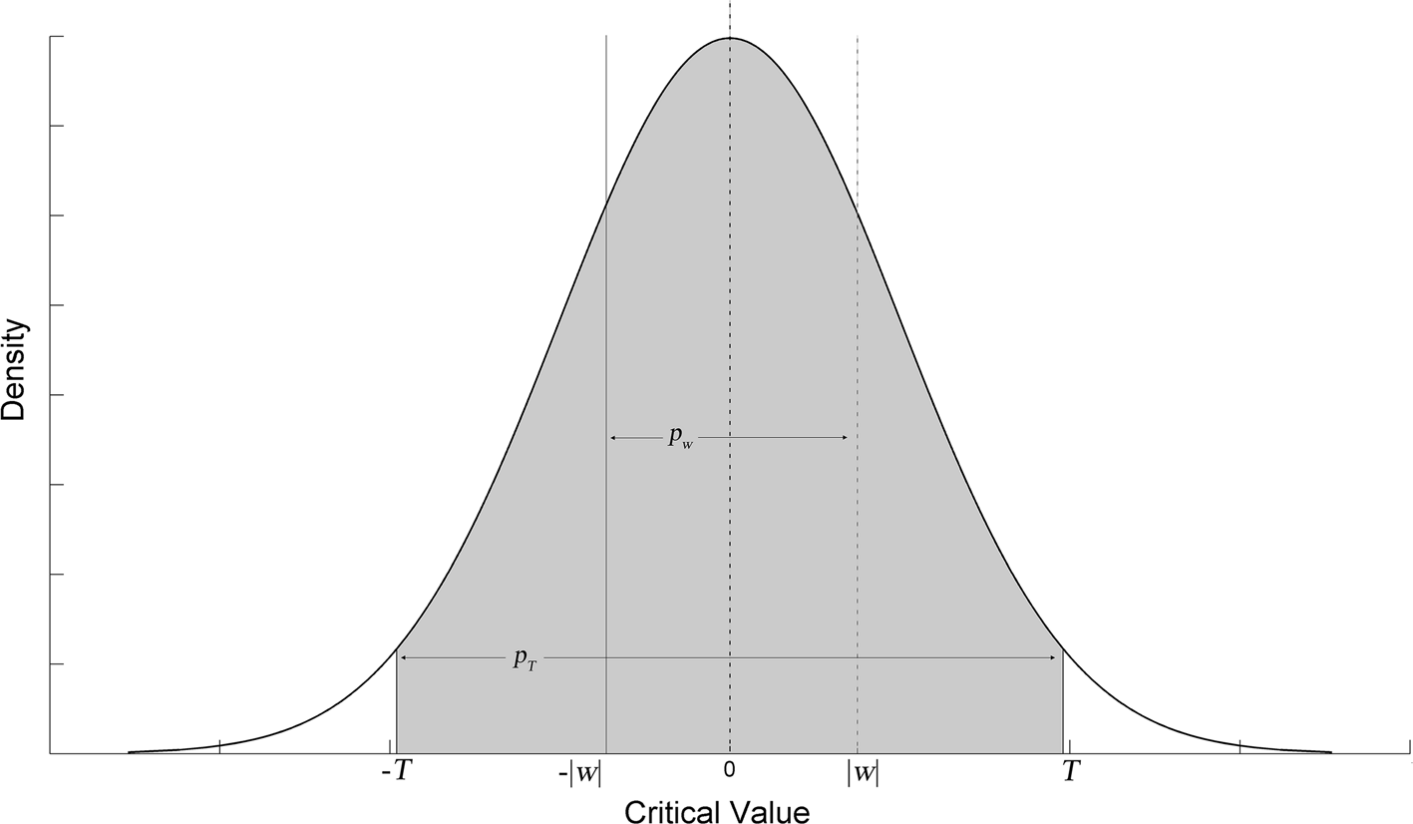
Illustration of the wavelet decomposition coefficients’ rejection probability and its comparison with threshold probability. For coefficient $w$, its corresponding rejection probability is $p_{w}$, which is the integration from $- \left| w \right|$ to $\left| w \right|$ with assumed Gaussian probability density function. $T$ is the threshold of $w$ and $p_{T}$ is corresponding threshold of $p_{w}$. If $w$ is artifact-free, the coefficients are spread around zero. Then the $p_{w}$ will be less than $p_{T}$. If $w$ is contaminated with movement artifacts, the coefficients will be discretely distributed and $p_{w}$ will be larger than $p_{T}$

$w_{jk}$ containing head movement will be larger and less possibly belong to the assumed $N$ distribution. It will be set to zero to suppress interference in reconstruction signals. Figure 2 illustrates this concept. Once the movement interferences in the brain EIT signal are suppressed, the resulted artifacts in the EIT images will be removed consequentially.

### On-line calculation for wavelet processing strategy

The Mallet method is usually used for discrete wavelet decomposition. It is based on multi-resolution analysis, wherein high- and low-pass filters are used recursively to achieve the projection of any signal on scale space using a scale function as an orthogonal basis, and on a wavelet space using wavelet function as an orthogonal basis. Normally, it is essential to acquire all data in advance to carry out wavelet decomposition. That is so-called pyramidal algorithm. All data are first read to construct the first layer of the pyramid and the values of the scale coefficient and wavelet coefficient of the first layer are calculated before calculating the second layer. The algorithm progresses layer by layer until reaching the required level. Therefore, data reconstruction can only be carried out after all decomposition is completed and wavelet decomposition and reconstruction are not carried out in real-time.

**Fig. 3:**
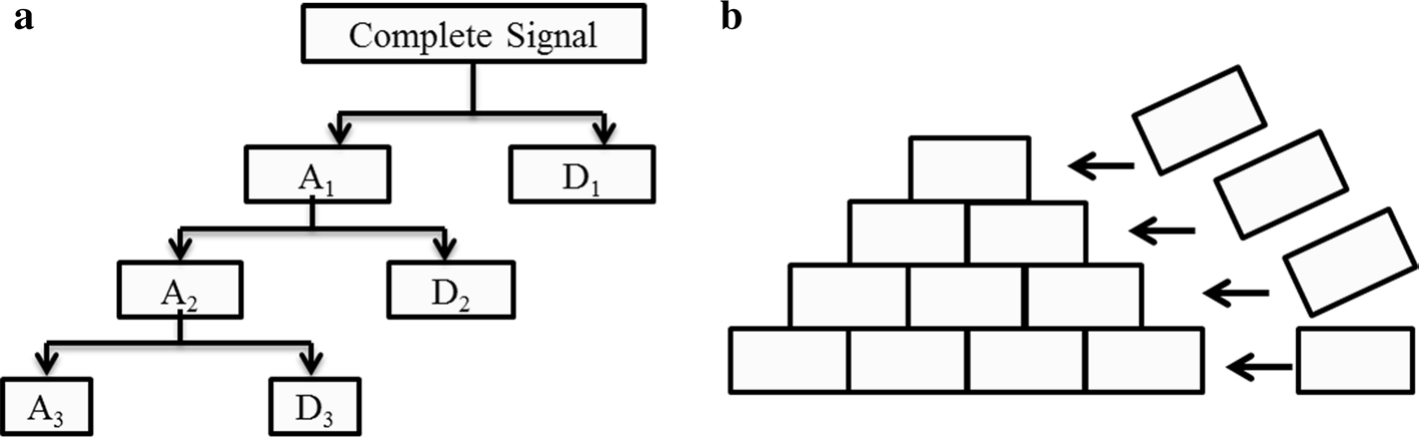
Comparison of traditional pyramidal decomposition and brick-laying decomposition. **a** Is the Mallet method to carry out wavelet decomposition with complete dataset. The decomposition is performed downward. **b** Illustrates the brick-laying method, the higher layer will be calculated once the lower layer offers enough coefficients. The computation is performed transverse and upward

In the theoretical analysis of multi-resolution, the support length of the scale function is not stipulated as finite length or infinite length. But in practical calculation, the support length of wavelet function is limited. Therefore, the decomposition filters are with finite length and only able to convolve limited data. So in the wavelet decomposition, there is no need to complete the calculation of the entire bottom layer before calculating the upper layer. The Mallet method can be modified [41]: we can perform once convolution in the higher decomposition level after twice convoluting in the lower level. The whole calculation is performed transversely and upward. The higher layer convolution can be calculated as long as the lower layer offers data long enough to perform one calculation. So we do not need to acquire all signal data to perform wavelet decomposition. Unlike the pyramidal algorithm, this kind of calculation is more akin to brick-laying. Figure 3 shows specific operations compared with conventional operations.

### Data processing parameter selection

The movement interference management based on wavelet method requires the confirmation of multiple parameters, including wavelet function, number of decomposition layers, screening threshold, and variance of the *priori* Gaussian distribution. The variance of the normal distribution is determined as the median absolute deviation in the decomposition coefficient sequence. The median absolute deviation has good robustness and is not sensitive to small numbers of outliers in the extraction sequence. In dynamic brain EIT measurements, head movement interferences present as individual outliers with high amplitude. Therefore, when median absolute deviation is used to estimate the variance, the result will not be sensitive to a few head movement interferences. The calculation for the distribution variance of the wavelet coefficient at each layer is [24, 42]:

15 $$ \tilde{\sigma }_{j} = \frac{{{\text{Median}}\left( {\left| {W_{j} } \right|} \right)}}{0.6745} $$

We need to determine the wavelet function based on the morphological and empirical choice. Considering that the majority of head movement interferences appear as spikes and limited filter length is required for real-time operations, db4 wavelet was used for signals’ decomposition and reconstruction. According to reports in which wavelet decomposition was used for signal processing, when decomposition reaches the fourth layer, processing results from further decomposition does not show any significant differences when compared with processing results at the fourth layer of decomposition. Differential probabilities with statistically significant differences were used as a reference for the selection of filtering threshold. When the corresponding adaptive probability of the current $w$ is greater than 90% (i.e., the probability that $w$ falls into the a priori normal distribution is greater than 90%), the source of $w$ does not include measurement signals that contain head movement interferences (i.e. $\alpha = 0.9$).

### Experiment validation design

To validate the feasibility of the proposed approach, we performed experiments with simulated data, phantom data and clinical measurement. The simulations and data processing were performed in Matlab version 2012b. All imaging reconstructions were implemented in self-developed software developed by C++.

Real EIT data were collected using an EIT system (FMMU-EIT5) [43]. This system uses a working frequency at 1–190 kHz, excitation current ranges from 500 to 1250 μA with measuring accuracy of 0.01%. The common-mode rejection ratio is over 80 dB. The image reconstruction algorithm was the damped least-squares algorithm developed by our research group [37]. We have used this system for previous clinical studies on brain EIT [44, 45]. In this study, we employed 500 μA and 50 kHz altering current to collect human data with the speed of 1 frame/s. All calculation were implemented on a Pentium G630 computer.

#### Simulation experiments

**Fig. 4:**
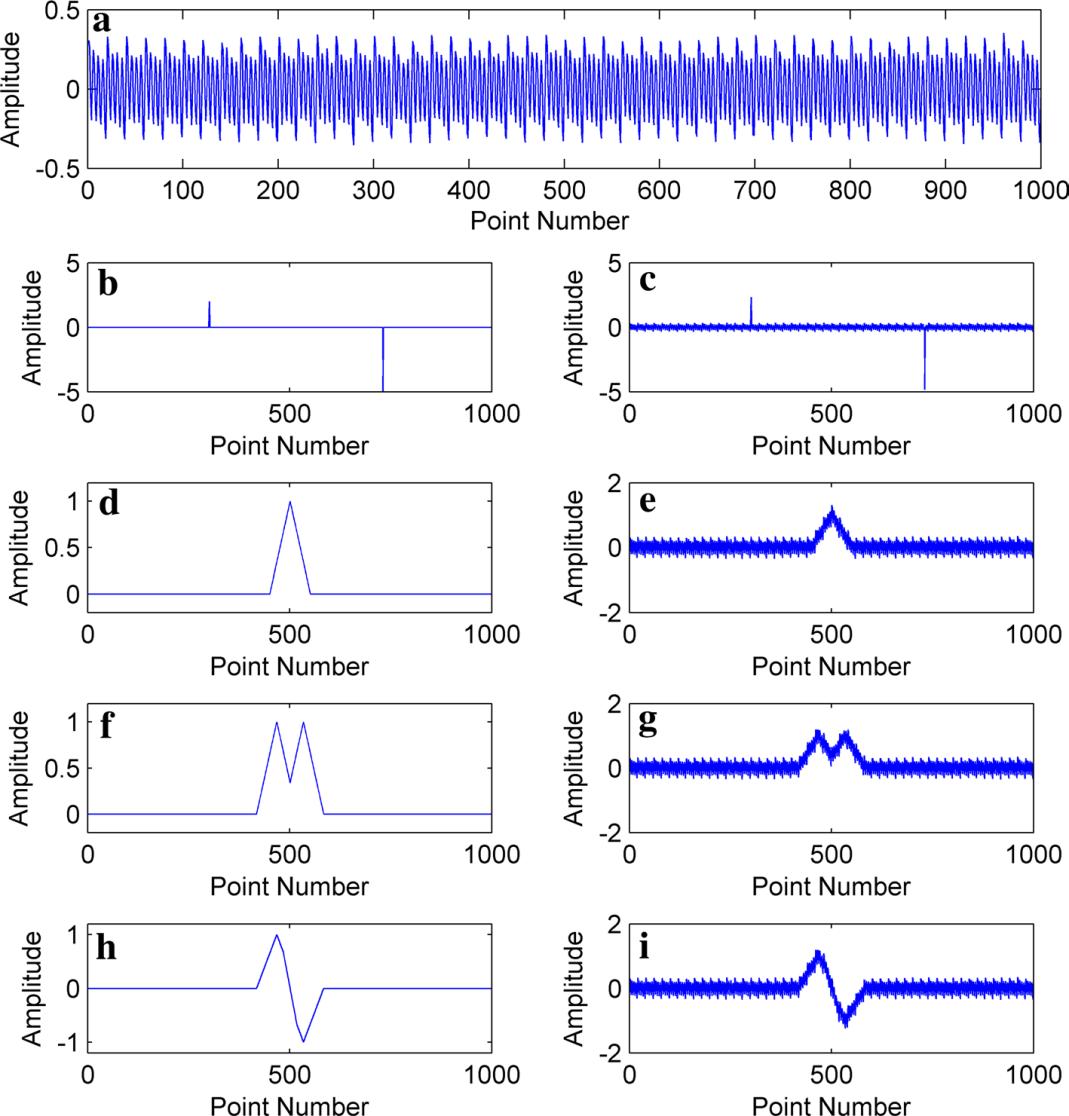
Simulated signal (**a**); different types of simulated head movement interference (**b**, **d**, **g**, **h**); signals obtained by adding (**a**) to movement (**c**, **e**, **f**, **i**)

The ideal brain EIT signals can be regarded as direct current signals. Therefore, while generating signals, we need the simulation containing similar typical changes of the EIT signal. We used the following equation to generate a segment of composite frequency sinusoidal signal with white Gaussian noise:

16 $$ x_{\text{simulate}} (t) = \frac{1}{3}\sum\limits_{i = 1}^{n} {\mu_{i} \sin (\omega_{i} t)} + 0.05\sigma (t) $$

where $$ n = 4, $$ $$ \omega = 2\pi f, $$ $$ \mu $$ represents the oscillation amplitude of the sine wave, $$ \sigma (t) $$ represents the Gaussian white noise, $$ \gamma $$ represents the amplitude of the Gaussian white noise, and the amplitude range for $$ x_{\text{simulate}} (t) $$ is $$ ( - 1,\;1) $$. The frequencies and amplitudes of the four types of sine waves used were (1 Hz, 0.6), (0.1 Hz, 0.9), (0.25 Hz, 0.9), (0.04 Hz, 1) and a total of 1000 data points were set, which were used to generate signals as shown in Fig. 4.

Spike signals simulating head movement interferences were added to the generated simulation signals to evaluate the consistency of post-processed and raw simulation signals. Evaluation was carried out using the three indicators of percent root difference (PRD), Pearson product–moment correlation coefficient (*r*), and coefficient of determination (*R*-square) [22].

17 $$ {\text{PRD}} = 100\% \times \sqrt {\sum\limits_{i = 1}^{N} {\left( {x(t_{i} ) - y(t_{i} )} \right)^{2} \left( {\sum\limits_{i = 1}^{N} {x^{2} (t_{i} )} } \right)}^{ - 1} } $$

18 $$ \begin{array}{*{20}ll} {r = \frac{1}{M}\sum\limits_{i = 1}^{N} {\left( {\frac{{x(t_{i} ) - \overline{x(t)} }}{{s_{x} }}} \right)} \left( {\frac{{y(t_{i} ) - \overline{y(t)} }}{{s_{y} }}} \right),\quad M = N - 1} \\ {{\text{with}}{\kern 1pt} {\kern 1pt} \;s_{x} = \sqrt {\frac{1}{M}\sum\limits_{i = 1}^{N} {\left( {x(t_{i} ) - \overline{x(t)} } \right)^{2} } } ,\quad s_{y} = \sqrt {\frac{1}{M}\sum\limits_{i = 1}^{N} {\left( {y(t_{i} ) - \overline{y(t)} } \right)^{2} } } } \\ \end{array} ,\quad M = N - 1 $$

19 $$ R{\text{-square}} = \frac{{\sum\nolimits_{i = 1}^{N} {\left( {y(t_{i} ) - \overline{x(t)} } \right)^{2} } }}{{\sum\nolimits_{i = 1}^{N} {\left( {x(t_{i} ) - \overline{x(t)} } \right)^{2} } }} $$

Here we consider $x(t)$ as original signal without movement interference. ${\text{PRD}}$ evaluates the consistency between $x(t)$ and $y(t)$ while $r$ and *R*-square evaluate the similarity between $x(t)$ and $y(t)$.

#### Physical phantom experiments

The physical phantom experiments were carried out on a resistor phantom representing a circular homogeneous medium and comprised of 120 resistors (1 kΩ) with 0.1% precision [46]. The phantom was connected to the data collection system through the SCIS-36 port [10]. Localized conductivity perturbation could be generated by operating the 16 push-type switches on the phantom. The finite element model for image reconstruction was a homogeneous circular mesh with 288 triangle elements. First, we acquired frames of EIT data without imaging target as the reference frames, and continued the data collection after pushing one switch to produce one conductivity perturbation as current frames. We simulated four different spike interference scenarios by adding different spikes on the current frame part of phantom data.

#### Human measured data experiments

The clinical data were acquired in the neurosurgery ICU of Xijing Hospital, Fourth Military Medical University, Xi’an, China. This study was approved by the human research ethics committee of the Fourth Military Medical University and informed written consent was obtained from the patient’s nearest relatives. In this scenario, two male patients were included. One patient suffered from head impact and had the risk of secondary injury. The other one was with cerebral edema and received mannitol dehydration treatment. There was no wound in the superficial scalp and all electrodes could be deployed. Both patients were conscious and lay on the sickbed. In the data collection process, no intervention was involved except for the patients’ movements themselves and nursing procedures performed by medical staff. Before data collection started, 16 rigorously disinfected cup-shaped electrodes were attached with conductive paste (Ten 20 conductive paste, Weaver and Company, Aurora, USA). Then, the electrodes were placed in an annular manner on the patient’s head. The finite element model for reconstruction was the same in phantom experiments.

## Results

### Validation with simulated data

**Fig. 5:**
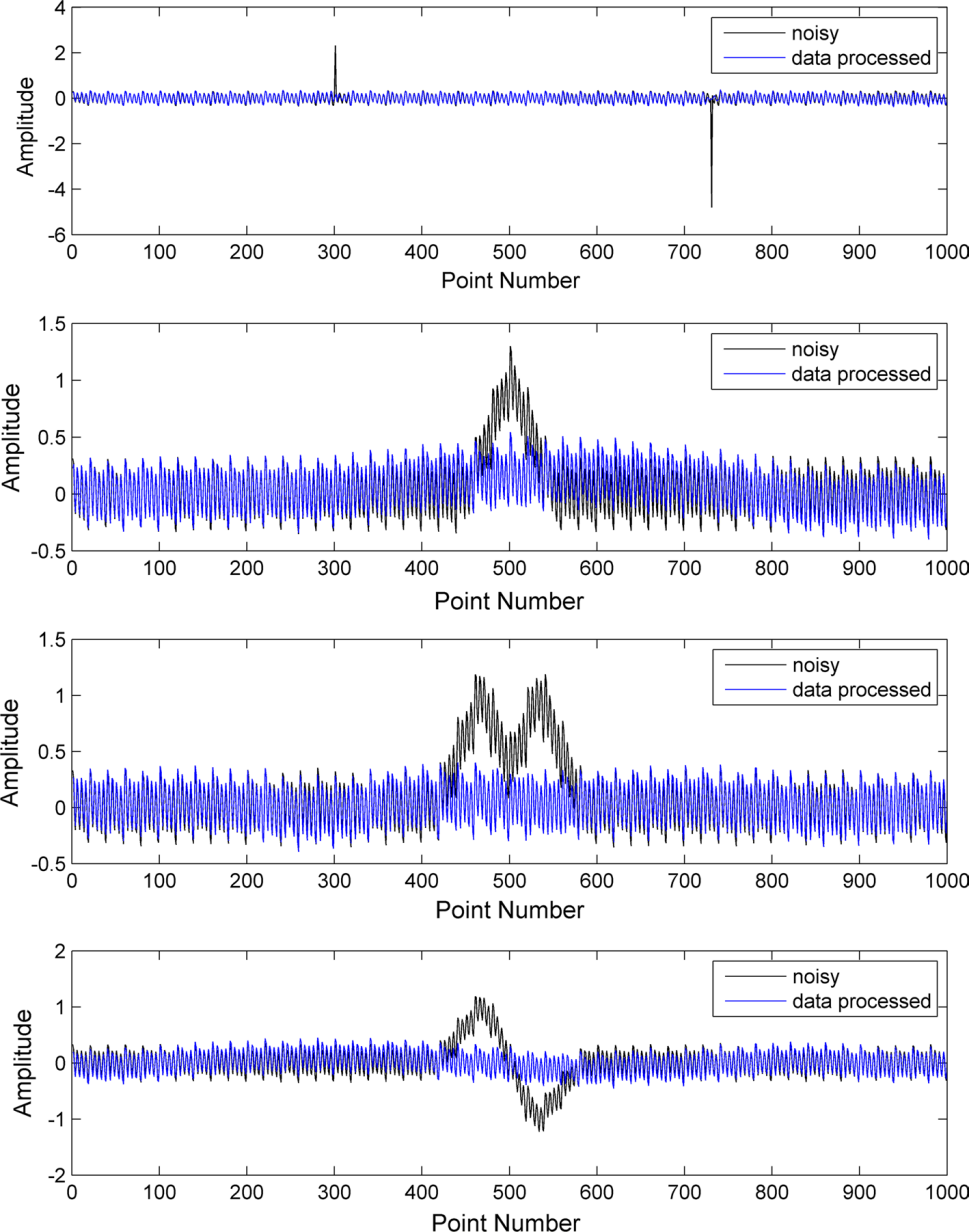
Results of the processing strategy on simulated signals. Each subfigure shows the comparison of signals before and after processing

The results of head movement processing method in simulation experiments are shown in Fig. 5 and Table 1. Db6 wavelet was used and the number of decomposition layers was 10 for all mixed signals. As shown in Fig. 5, we can see that the four kinds of simulated spike interferences were effectively restrained in the presence after processing. Table 1 shows the quantitative indicators of post-processed signals. All indicators show improvement in data quality. PRD was increased by at least 48% and *r* advanced 43% in average. In type 1 and type 2 simulation, the *R*-square had sharp promotion, which indicates the goodness of the processed signal.

**Table 1:** PRD, r and *R*-square values for comparison between the simulation signals with simulated interference (type 1, type 2, type 3 and type 4) and with reduced simulated interferences (type 1-processed, type 2-processed, type 3-processed, type 4-processed)

	PRD	*r*	*R*-square	∆PRD	∆*r*	∆*R*-square
Type 1	0.9096	0.7360	0.1726	73.28%↓	31.81%↑	445.18%↑
Type 1-processed	0.2430	0.9701	0.9409			
Type 2	0.9753	0.7157	0.0488	48.15%↓	24.08%↑	1426.08%↑
Type 2-processed	0.5057	0.8880	0.7442			
Type 3	1.3657	0.5904	0.8650	80.16%↓	63.23%↑	7.12%↑
Type 3-processed	0.2709	0.9637	0.9266			
Type 4	1.3240	0.6043	0.7531	73.28%↓	53.44%↑	12.12%↑
Type 4-processed	0.3945	0.9273	0.8444			

The ∆ values correspond to percentage changes comparing the data with and without wavelet processing

### Validation with resistor phantom data

**Fig. 6:**
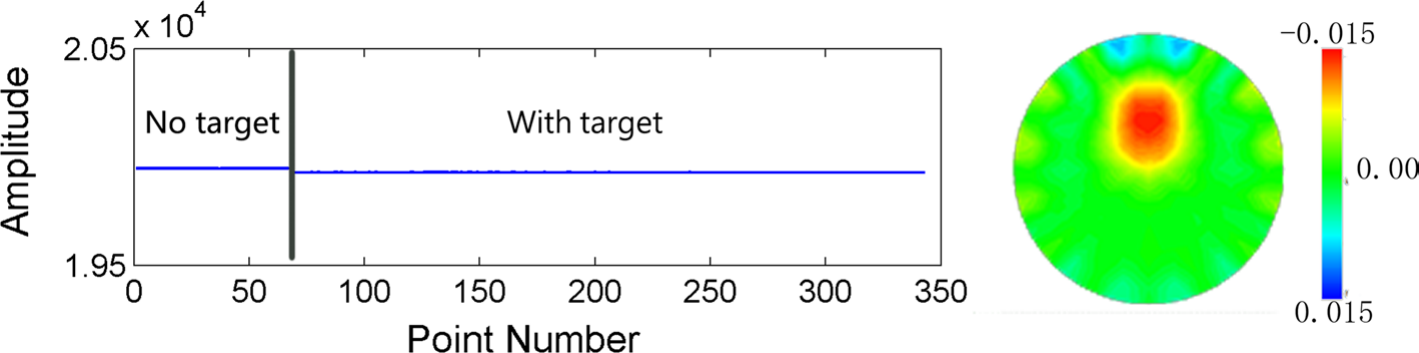
Mean values (left side) and the normal EIT image with imaging target (right side) of the resistor phantom data

Figure 6 shows the mean values of continuous phantom data and the EIT image containing conductivity perturbation target. The length of the data was almost 350 frames. Db6 wavelet with 6 decomposition layers was employed to implement the processing.

**Fig. 7:**
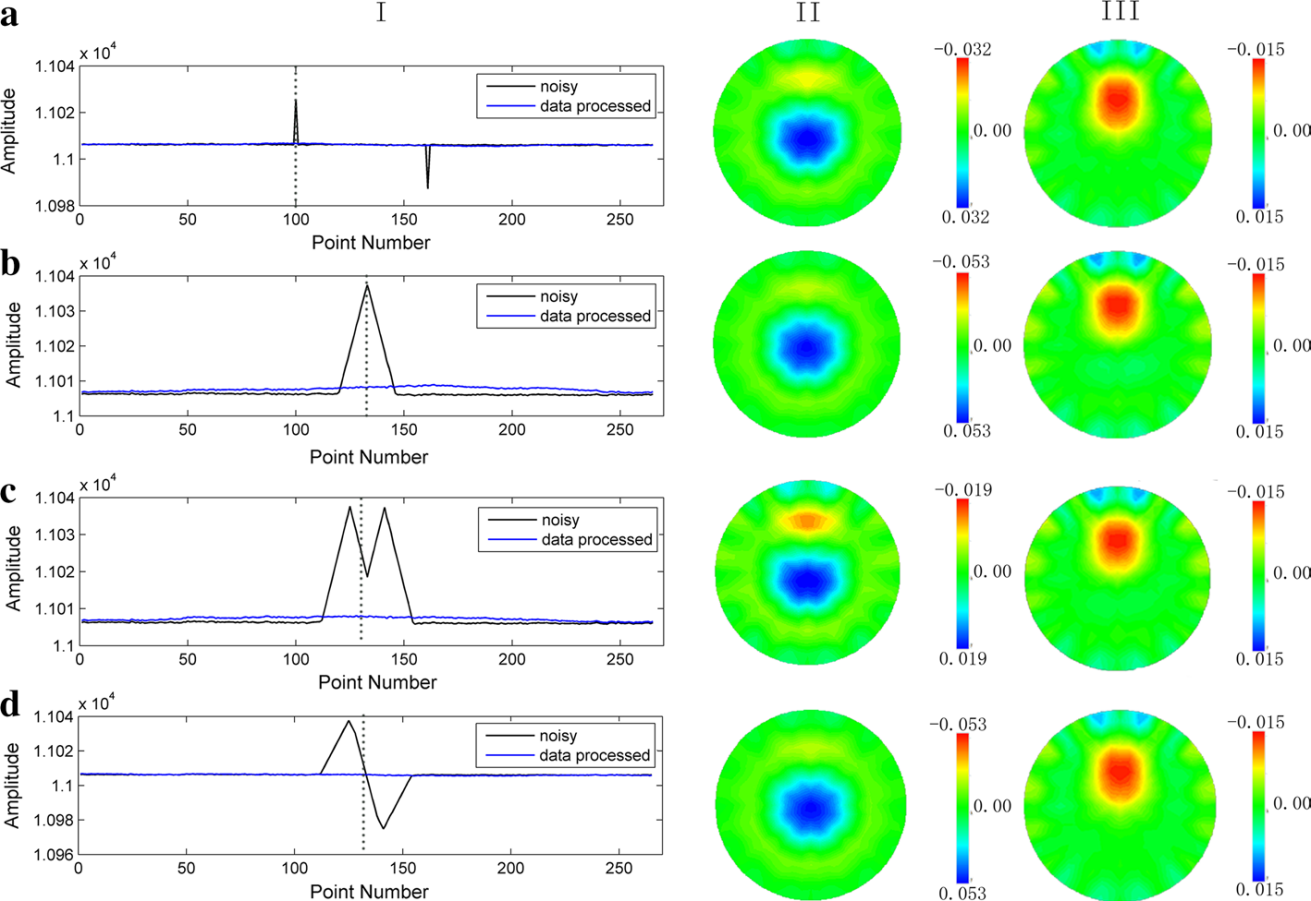
Results of wavelet processing experiments on resistor phantom. Rows A–D are four different scenarios of spike interference added in normal data collected on resistor phantom. Column I shows the mean value of data frame before and after processing. Column II presents the EIT images contaminated by added spikes. In Column III is the EIT images reconstructed with processed data by proposed method. In Column I, the dashed lines mark the current frame of the EIT images in Column II and III

The processing results were presented in Fig. 7. We added four types of spike contamination to the perturbation-included part of phantom data and testified the effectiveness of the proposed method. Rows A–D correspond to the four different scenarios. Column I–III, respectively, illustrate the comparison of mean values as well as imaging results of phantom data before and after processing by the proposed approach. From Column I, we can see that the spikes decreased greatly in time series. The artifacts introduced by spikes are shown in Column II. The original target was nearly lost in the EIT images. Compared to the images in Column II, the artifacts were removed and original perturbation targets were restored in the EIT images in Column III after processing.

### Validation with human measurement data

**Fig. 8:**
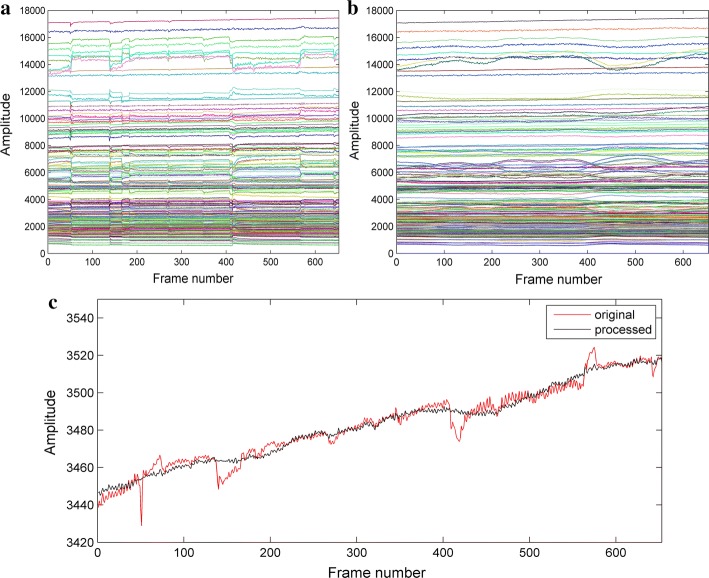
Human measurement data processing results. **a** Data of 192 channels before processing. **b** Data of192 channels after processing. **c** Mean value comparison of 192 channels’ data before and after processing

**Fig. 9:**
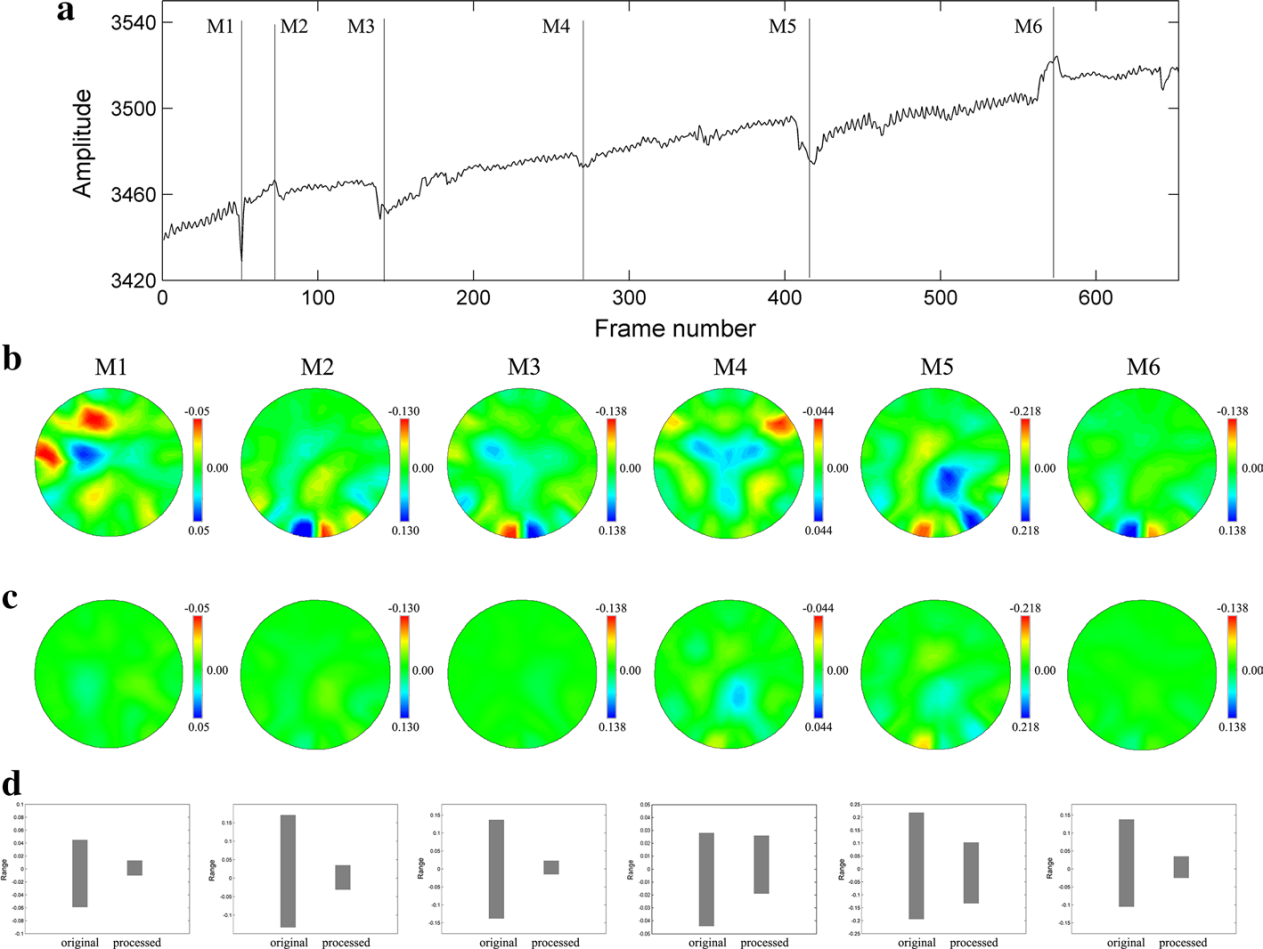
Comparison of reconstructed images with the original data and processed data. Row A is the mark of head movement occurrence, M1–M6 is the sequence number of head movement. Row B is the images reconstructed with original data. Row C is the images reconstructed with processed data. Row D is the comparison of reconstruction value range before and after data processing

Figure 8 shows the processing results of human measurement data. One segment data of clinically measured data containing 650 frames long was selected, which included several observed head movement interferences. Db4 wavelet with 4 decomposition layers was used for data processing. Figure 8a shows the waveforms of 192 channel data in time series before processing while Fig. 8b shows the data waveforms after processing. By comparing the signal waveforms in Figs. 8a and 9b, it could be seen that the head movement interferences were reduced considerably in all channels. To demonstrate the results more specifically, we made a comparison of the mean values before and after processing. Figure 8c illustrates the comparison results of mean values and the single channel spikes reflecting in mean values were restrained. Therefore, our method is able to manage the spike type of movement at signal level.

Considering EIT, an imaging monitoring technique, we testified the feasibility of proposed processing method by reconstructing the EIT images with processed data. Figure 9 shows the imaging results before and after data processing. In Row A, labels M1–M6, respectively, indicate the time locations that movement interference happens. Considering the processing expectation was to inhibit head movement interferences, which only exist in current frames, we selected one frame of data before movement occurrence as reference frame. Following this principle, EIT images in Row B are the reconstructed results contaminated by movement artifact. Columns M1–M6 represent that the images in this column correspond to different movements marked in Row A. The EIT images in Row C are corresponding outcome after proposed management. Row B shows significant artifacts introduced by head movement interferences in the reconstructed images. After signal processing by the proposed method, the artifacts are greatly reduced in corresponding EIT images of Row C. Row D indicates the variation range of reconstructed values. Because the movement usually leads to outliers in the signal, the reconstruction value will be also amplified if movement interference happens. Therefore, through the downscaling of reconstruction value variation in row D, the artifacts in reconstructed images were suppressed effectively. All results above prove that the proposed method is able to restrain head movement interferences at signal level and image level.

### Evaluation of time and storage cost

**Fig. 10:**
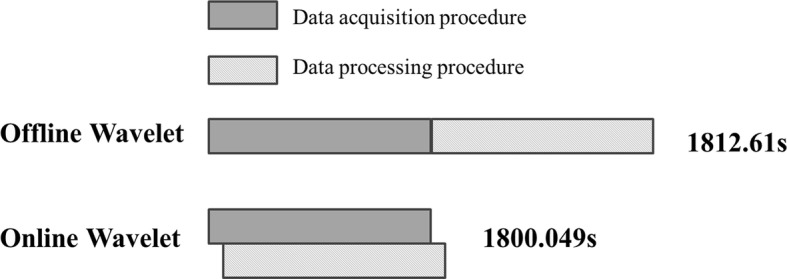
Comparison of calculation time and storage cost with online wavelet method and offline wavelet method

Figure 10 shows the time cost for real-time calculations in wavelet data processing. As data acquisition and data processing were carried out simultaneously, total time was significantly decreased. While the data acquisition interval is 1 s and time required for wavelet processing is far lower than 1 s, online processing is feasible.

According to the algorithms used for pyramidal method and brick-laying method, we set that wavelet data processing requirements for signals with a length of *A*, one layer of decomposition, wavelet filter length of *N* has a total number of data stored of:

20 $$ {\text{Offline: }}A + \sum\limits_{i = 1}^{i = l} {\frac{A}{{2^{{{\text{floor}}(i/2)}} }}} $$

21 $$ {\text{Online: }}1 + \sum\limits_{i = 1}^{i = l} {\frac{1}{{2^{i - 1} }}\left( {\left( {l + 1} \right)N + 2^{l} \left( {\frac{N}{2}} \right) + \left( {2 - 2^{l + 1} } \right)} \right)} $$

Calculating the storage cost of the human measurement data in Fig. 9, the offline processing requires $1.78 \times 10^{3}$ storage units while on-line processing only requires 130 storage units. Therefore, storage space requirement is greatly reduced in on-line calculation.

## Discussion

This paper analyzed the problem of head movement during dynamic brain EIT monitoring in clinical environments, and we managed the head movement interference based on the distribution characteristics of wavelet decomposition coefficients. In simulation experiments, the spikes which simulate head movement interferences were suppressed effectively after being processed by the proposed approach. The correlation between processed signal and raw signal was significantly increased, which could be concluded through the quantitative indicators such as PRD, correlation coefficient and *R*-square. After that, we simulated conductivity perturbation on the resistor phantom and collected the phantom data with spike interferences. The proposed method could remove the artifacts and restored the imaging target at the same time. To testify whether the proposed method was able to improve the clinical practicability, we collected human data in the ICU for validation. After processing, the continuity brain EIT signal was restored and movement artifacts were removed from the reconstructed images. Besides, we employed brick-laying calculation to adapt to dynamic brain EIT monitoring.

In simulation experiments, we used different types of spikes to simulate head movement interferences in the brain EIT signal. This choice is based on scenario analysis and actual data observation. By comparing the real movement signal, it can be seen that the spikes could well conform to the character of head movement interferences. In phantom data experiments, we demonstrated the efficacy of the proposed approach while there was simulated perturbation included in the measurement. The target information was well restored and the artifacts were suppressed effectively. In human data experiments, we could not get pure signal without movements at the same time based on existing equipment. In this way, quantitative indicators could not be calculated to measure the processing effect of real human data. Unlike the simulated signal, there were more components except for noises and spikes in the real human data, like the step-type interference, which made the processed curve less match compared with the simulations. But the further image results demonstrate the feasibility of the proposed approach. Here we only used artifact-free images to demonstrate the processing effect. There should be no voltage variation caused by intracranial pathophysiological change between the temporary reference frame and the current frame, which were a few seconds apart. The imaged targets were introduced by spike-type interference were well removed after data processing.

**Fig. 11:**
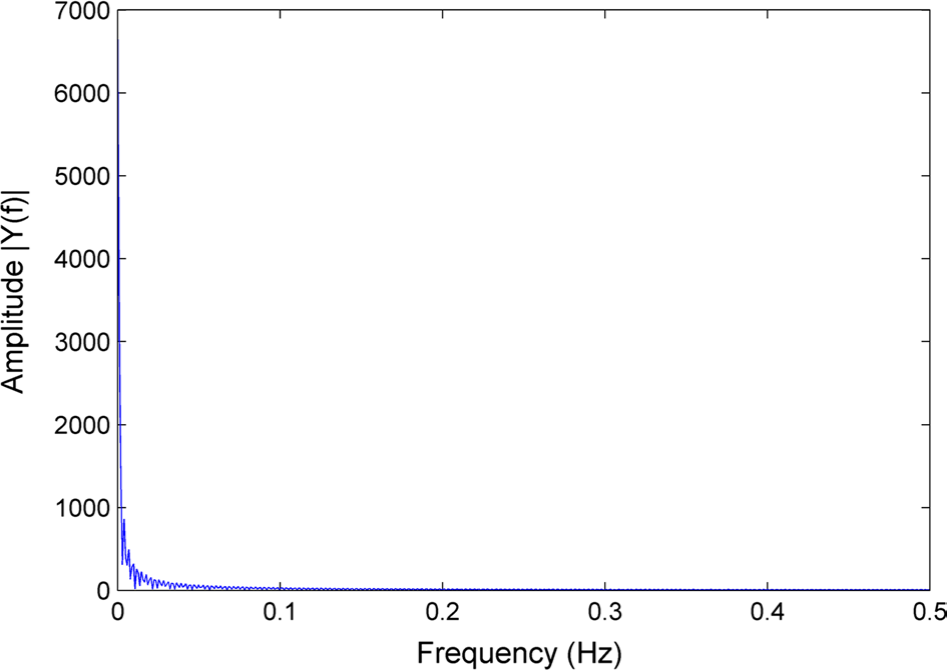
Frequency–amplitude spectral of brain EIT data contaminated with movement artifacts. The energy of the whole power almost concentrated in the DC segment and there is no remarkable difference in frequency domain for us to employ data processing

In previous studies, it was found that brain EIT signals showed continuity with slow changes in time series. On one hand, this is due to data acquisition speed being 1 frame/s. On the other hand, since the resistivity of the skull is high, the boundary voltage changes caused by intracranial impedance changes are relatively small in amplitude. In addition, considering that intracranial pathophysiological changes that cause impedance changes also require some time, brain EIT signals nearly become direct current (DC) signals during a segment of frequency domain analysis. The head movement interferences appear as relatively drastic changes in amplitude in an instant. Meanwhile, it takes 10 min or more time for intracranial impedance variation to result in such change in boundary voltage. Intuitively, it can be seen that normal signals and head movement interference signals show differences in frequency. However, compared with the baseline of measurement, these changes are still small in amplitude. As shown in Fig. 11, the frequency band including all head movement interferences in the entire EIT signal was very narrow, and the energy of the signal was almost concentrated at the DC segment. Thus, it is not convenient to obtain prior information of movement interferences through the use of conventional digital filter methods for processing. Therefore, the frequency domains of head movement interference signals and normal signals can be considered being mixed, and only show significant distinction in the time domain. This makes characteristic time–frequency analysis methods more suitable to process the interferences. Besides, the traditional signal processing method, such as low-pass filter, separates interferences from normal signal of brain impedance variation in frequency domain. If the smoothing effect of low-pass filter is good, it will inevitably cause the signal to be blurred, which means suppression of normal biological signal. In this case, the effect of processing is at the expense of losing useful signal components. But, in signal processing by wavelet decomposition, we decompose the various frequency components in the signal into bands that do not overlap each other and only managed the parts contaminated by movement interferences. So the normal signal of brain impedance variation is left with minimum suppression.

The processing method selected in this study was based on the priori distribution information of the wavelet decomposition coefficient. If wavelet coefficient is “sparse”, i.e., the wavelet coefficient is composed of large number of small coefficients and a few large coefficients, then the Gaussian mixed distribution with two-component zero mean can effectively fit the actual wavelet coefficient distribution [47]. The differences in these two Gaussian distributions lie in the variance. The distribution with a large variance will have a low a priori probability, which represents few large coefficients; while the distribution with a small variance will have a high priori probability, representing a large number of small coefficients. The overall brain EIT detection signals are relatively smooth and slow-changing, and their wavelet decomposition coefficients can satisfy the “sparse” requirement. In addition, the characteristics of these signals are similar to previously studied body movement interference signals. Therefore, the use of wavelet decomposition in this domain is reasonable [24].

During dynamic brain EIT continuous monitoring, head movement interferences will change electrode–scalp contact impedance, which will appear as changes in boundary voltage and ultimately affect image reconstruction. In previous reports, researchers have focused on the effects of contact impedance on the reconstructed images. However, such contact impedance changes often originate from conductivity changes due to electrochemical changes at the electrode-conductive paste–scalp contact layer. In the reconstruction process, we demonstrated that conductivity changes at the electrode contact layer are actually equivalent to impedance changes at one area in the reconstruction field. However, reconstruction algorithms themselves cannot differentiate whether voltage changes originate from the contact layer or inside the field. Limited by the FEM use during reconstruction, these surface contact changes are regarded as impedance changes inside the field and appear as reconstruction artifacts in reconstructed images [48, 49]. Current researches mainly modify imaging algorithms with complete electrode models to isolate contact impedance changes and their effects on specific elements in the finite element model [48, 50, 51]. However, this kind of processing strategy does not consider the influences of contact impedance changes on excitation current, and can only be used to process contact impedance problems in which the magnitude of such change is smaller than head movement interferences. Previously, other studies on processing of more severe body movement interferences were targeted at extreme situations in which there is saturation or distortion of excitation current, or inability to normally enter the field. Therefore, data-abandoned processing strategies were used for affected measurement values. In the clinical environment, the movement interferences in the middle of the two scenarios are more common. The measurement data can still reflect the actual boundary voltage to some extent, but contains head movement inference component.

Our method employed the wavelet processing strategy proposed by Molvavi. Unlike the previous study, on-line calculation was carried out in the actual implementation, improving its practicality. In Molvavi study, the reason why this method could not be used in real-time was due to the variance estimation strategy employed. During dynamic brain EIT measurement, when electrodes fully contact the scalp, the corresponding measurements are generally similar. Therefore, it is feasible to use variance values calculated from past data as priori distributed variance values. Chen et al. [23] proposed a processing strategy similar to Molvai. However, they directly converted the probability threshold into the screening threshold for specific wavelet decomposition coefficient to deal with motion artifact. In addition, they set the upper and lower limit threshold values and could suppress noise in signals by processing body movement interferences. In dynamic brain EIT scenarios, noise that is simultaneously present in data from the current and reference frames are already inhibited during the imaging process and there is no need to care for such noise. The conversion of probability to specific threshold number increased the calculation, especially such operation results were uncertain. Compared with other methods, that method does not need the input of any reference signal and reduces the requirement for priori information to the minimum. Compared with traditional wavelet processing methods that used fixed thresholds, the use of an adaptive probability threshold can achieve specific elastic changes in coefficient threshold and has stronger adaptability.

Our processing strategy is mainly aiming at spike-type interferences in clinical practice. Patients’ movements or medical staffs’ operations will change the contact status of the electrodes. But the electrode’s contact will restore to its initial status with the elastic shrinkage of bandages used for fixing. In this way, head movement interferences will present as uneven spikes in the signal. The characteristic of the head movement interference is no baseline alteration. The movements are discrete points in the time series. The wavelet method isolates the frequency-domain aliased interferences through its time–frequency analysis function. If the baseline is affected by movement disruption of electrode status, the processing results will be declined. That is because the data after discontinuous points show little difference compared with the normal signal in wavelet domain while it still contains baseline alteration. Therefore, the processing results with human data are a little inferior in waveform compared with the simulations. There is a special brain EIT application case, epileptic seizures imaging, where the spike-type signal is monitoring target [52, 53]. The seizures are occur in tens of seconds and more like movement artifacts, which makes the seizures difficult to be separated from the real movement artifacts. Although epileptic seizures can be detected by brain EIT theoretically, it is not ready for actual clinical application because of this limitation [54]. Our main object is also not epileptic seizures monitoring, so the situation that secondary seizures mixed with movement artifact is not included and considered in our study.

Besides, our scope of application with the proposed wavelet method is to manage the movement interferences in the long-term brain EIT monitoring scenarios [44, 45]. Because of the skull’s high resistance, the boundary voltage variation from intracranial impedance change could be only 1–3%, in our previous twist drill drainage and clinical dehydration treatment experiments. Considering the time cost of treatment (for example, the dehydration needs at least 60 min, the twist drill drainage needs tens of minutes), the monitoring procedure appears as a flat and slow-varying signal. The head movement introduces interference into the normal signal through changes in current field distribution near the affected electrodes. So the head movement can be considered as one kind of impedance variation in the field. The resulted spikes may not be larger in the amplitude but more acute gradient compared with the normal signal in the same time. In addition, we applied data collection for 1 frame/s. This data collection speed is too slow to acquire much faster changes like blood flow or other physiological activity. In this way, we do not consider these non-interested signals in our research. There is another concern that we could just exclude the data contaminated by movement, just like the EEG monitoring, where it is sufficient to label portions of the signal with movement artifacts. If we target the EIT monitoring at the pathological process which may be occurring over an hour, the exclusion strategy is acceptable in such cases, especially in offline data analysis. However, we are not able to know how soon or how long the pathological process will be happening beforehand. Meanwhile, we hope to make the EIT monitoring more practical in clinical monitoring by removing the artifacts with online management. Therefore, we prefer to employ the online processing method to extend its potential application and keep as much data as possible.

While using the wavelet method to deal with head movement interference, we need to determine the variance of the Gaussian distribution and the rejection probability. To acquire the variance of the Gaussian distribution, it is necessary to acquire all wavelet decomposition coefficients of the measurement. However, this is not possible during dynamic brain EIT monitoring. Therefore, the priori variances used during on-line processing are empirical values. Although validation experiments using clinical measurement data have obtained satisfactory results for empirical variances, the versatility of this method requires further validation. In addition, the rejection probability threshold determines the intensity of head movement interferences that require processing. Currently, to simplify the calculation, all 192 channels in EIT signals use the same threshold. However, the intensity of head movement interferences in all channels may not be identical. Therefore, more optimization of choosing parameters will be done in further study.

To manage the movement interference, there are some ad hoc algorithms, for instance, the compensation strategy based on spline interpolation proposed by Scholkmann [22]. Movements were detected by moving standard deviation and corrected by empirical correction value. Although the processing idea is relatively simpler, the reliability of the chosen correction value is a problem. The strategy of determining correction value limits its application in real-time processing. As for the PCA method, it applies the similar idea to separate different level of signal components based on Gaussian distribution like the wavelet decomposition does [55]. The main concern of the component lies on two aspects: first, the principle component reserved to reconstruct the signal. We need to distinguish the principle component which can represent the normal signal through eigenvalue or other mark value. While the data keep flowing, the component choice may need to be altered. Second, the real-time capability of the processing procedure. Usually, the PCA is employed when all data are known. So if we want to apply the PCA to restrain the head movement in brain EIT signal, more modification on on-line calculation of PCA indicators and the correction of the eigenvector estimation from theoretical level, which may make the strategy unable to act its advantage in simpler and quicker processing.

In addition, the data preprocessing time for brain EIT is significantly different from processing methods used for other bioelectrical signals. After data processing, the feasibility of the results is ultimately dependent on the reconstructed images. Therefore, brain EIT signal preprocessing has more stringent requirements compared with preprocessing of other bio-signals. As such, we not only require quality evaluation of time series signals, but also further joint evaluation indicators for time series signal quality and reconstructed figure results. Mamatjan et al. [56] proposed quantitative indicators for evaluating data quality by connecting acquisition data quality obtained from electrodes with image quality, and evaluating data from an image perspective. The method provided an overall assessment of the whole dataset. In further study, we will try to establish indicators to connect the image quality to single data channel to acquire head movement interference in the brain EIT measurement and quantitatively estimate the processing effect from image point.

The wavelet processing method is suitable for all time-difference EIT application theoretically. In this study, we implemented data collection with working frequency at 50 kHz and acquiring 1 frame of data per second. There is some time-difference EIT data measured at different working frequencies. And the time-difference data set is composed of averaged frames data to improve the accuracy of measurement. Therefore, we hope to try the proposed method on time-difference EIT data under other collection conditions and test its broadness in future work.

## Conclusion

In clinical long-term dynamic brain EIT, head movement interference is a common occurrence and will lead to image artifacts. This paper offers an on-line strategy to process the contaminated measurement through wavelet decomposition. We used mixed Gaussian distribution to describe the wavelet coefficients and detected coefficients corresponded to movement signal by distributed probability to process. Besides, modification was carried out to realize on-line processing for dynamic brain EIT monitoring. While the feasibility of this method is proved by experiments with reduced movement component, it provides an idea for data processing of brain EIT and lays a foundation for further research in data processing of brain EIT.

## Abbreviations

EIT: electrical impedance tomography
FEM: finite element model
PCA: principle component analysis
ICA: independent component analysis
PRD: percent root difference
DC: direct current
